# Occlusal Splint Therapy Combined with Cranio-Temporomandibular Kinesiotherapy in Patients with Temporomandibular Disorders—A CBCT Study

**DOI:** 10.3390/life12122143

**Published:** 2022-12-19

**Authors:** Manuela Tăut, Smaranda Dana Buduru, Daniel Tălmăceanu, Alina Ban, Raluca Roman, Daniel Leucuța, Ioan Barbur, Aranka Ilea

**Affiliations:** 1 Department of Oral Rehabilitation, “Iuliu Hațieganu” University of Medicine and Pharmacy, 400029 Cluj-Napoca, Romania; 2 Department of Prosthetic Dentistry and Dental Materials, Prosthetic Dentistry, “Iuliu Hațieganu” University of Medicine and Pharmacy, 400029 Cluj-Napoca, Romania; 3 “Stomestet” Dental Clinic, Department of Oral Surgery, 400372 Cluj-Napoca, Romania; 4 Department of Maxillo-Facial Surgery and Radiology, Dental Radiology, “Iuliu Hațieganu” University of Medicine and Pharmacy, 400029 Cluj-Napoca, Romania; 5 Department of Medical Education, Medical Informatics and Biostatistics, “Iuliu Hațieganu” University of Medicine and Pharmacy, 400029 Cluj-Napoca, Romania; 6 Department of Maxillo-Facial Surgery and Radiology, Surgery and Maxillo-Facial Implantology, “Iuliu Hațieganu” University of Medicine and Pharmacy, 400029 Cluj-Napoca, Romania; 7 Department of Maxillo-Facial Surgery and Implantology, Faculty of Dental Medicine, “Iuliu Hatieganu” University of Medicine and Pharmacy, Cardinal Iuliu Hossu Street, No 37, 400029 Cluj-Napoca, Romania

**Keywords:** CBCT, TMD, occlusal splint, kinesiotherapy, condylar ratio

## Abstract

Occlusal splints are used as a non-invasive treatment for patients diagnosed with temporomandibular disorders (TMD). Another non-invasive treatment option for TMD patients is kinesiotherapy, which may be used alone or in conjunction with occlusal splint therapy. The aim of this study was to evaluate the changes in the intra-articular spaces of the temporomandibular joint (TMJ) after occlusal splint therapy combined with cranio-temporomandibular kinesiotherapy in TMD patients. Twenty-four patients (*N* = 24) diagnosed with TMD according to Research Diagnostic Criteria for Temporomandibular Disorders (RDC/TMD) were included. Cone beam computed tomography (CBCT) images were taken before and after treatment. The anterior, superior, posterior, and medial joint spaces were measured on the CBCT images, and the condylar ratio was calculated using Pullinger and Hollender’s formula. Additionally, the thickness of the glenoid fossa (GFT), condylar medio-lateral widths (MLW), and condylar height (HCo) were measured. The condylar ratio was significantly higher after treatment (*p* = 0.049). The changes in dimensions of the anterior, superior, posterior and medial joint spaces were not statistically significant after treatment. No statistically significant differences were found in the dimensions of the GFT, MLW, and HCo post treatment. The condylar position did not shift anteriorly in a statistically significant way after occlusal splint therapy combined with cranio-temporomandibular kinesiotherapy in TMD patients.

## 1. Introduction

For both volumetric and linear measurements of temporomandibular joint (TMJ) structures, cone beam computed tomography (CBCT) has been demonstrated to be accurate and reliable, specifically designed for imaging in the oral and maxillofacial fields [1]. Moreover, the accuracy of CBCT linear measurements was found to be within 0.3 mm, which allowed for clinically accurate and reliable measurements of the craniofacial complex, even in the presence of soft tissue [2]. Another study conducted on 28 dry skulls showed that variation in skull orientation during image acquisition did not significantly influence linear measurements [3]. However, considering the small intra-articular TMJ spaces and the importance of the collected data for the final diagnosis, several factors should be taken into consideration both during and after 3D data acquisition: the type of CBCT machine, the head position and the reorientation of the skull after segmentation, the field of view (FOV), and the reliability and repeatability of identification of the reference slices for measurements [4].

Occlusal splints are indicated as a viable non-invasive treatment for patients diagnosed with temporomandibular disorders (TMD). Occlusal splints allow the lateral pterygoid muscle to relax and thus to center the condyles into glenoid fossa (GF). Together with the anterior deprogrammer and the leaf gauge, occlusal splints are frequently used for the treatment of dysfunction signs and symptoms such as muscle pain, TMJ pain, clicking, crepitus and restricted mandibular movement [5,6]. Although some authors highlighted the benefits of occlusal splints, others claimed that their effectiveness was modest and that more research is needed [7]. A meta-analysis, evaluating short- and long-term effects of occlusal splints for treatment of TMD, revealed short-term benefits in pain reduction and in increasing the maximum mouth opening. For long-term follow-up, the effects seemed to be the same as those of other therapeutic options [8]. According to another meta-analysis of 52 randomized controlled trials (RCTs), the most effective short-term (≤5 months) treatment option for painful temporomandibular disorders of muscular origin was manual therapy (83.5%, low-quality evidence), which was followed by ozone therapy (75.7%, very low-quality evidence), counseling therapy (71.2%, moderate-quality), and occlusal splints (71.7%, moderate-quality evidence) [9]. Al-Moraissi et al. [10] conducted another meta-analysis of 48 RCTs which revealed that there was a significant decrease in pain intensity in arthrogenous TMDs after hard stabilization splint therapy (52.9%, moderate-quality evidence) and a significant decrease in pain intensity in myogenous TMDs after hard stabilization splint therapy (59.7%, moderate-quality evidence).

Another non-invasive therapeutic approach for TMD patients is kinesiotherapy. Treatment of TMD has been found to be beneficial using manual therapy. It can be utilized independently or in addition to occlusal splint therapy [11]. Rocabado’s 6 × 6 exercises showed to be effective in improving masseter muscle elasticity in the reduction of pain score [12,13].

The position of the condyles in the GF, meaning the concept of centric relation (CR), was introduced in dental literature almost one century ago and its definition has changed over the years. The original definition changed as a result of several, frequently conflicting research findings [14]. This has ultimately led to the currently accepted format in the latest Glossary of Prosthodontic Terms, Ninth Edition and it reads as follows: “CR is the maxillomandibular relationship, independent of tooth contact, in which the condyles articulate in the anterior-superior position against the posterior slopes of the articular eminences” [15]. The current definition of CR, however, does not take into consideration the position of the articular disc. According to Dawson [16], the CR position is the most comfortable and stable position of the jaws, in which the joints can be subjected to load without causing discomfort. Other authors indicated that CR is a range of normal positions, several CR positions being acceptable [17,18].

Another study suggested that position of condyles within the GF has been associated with TMD and, consequently, the optimization of condylar position is indicated using therapeutic procedures such as muscular deprogramming and occlusal equilibration [19]. According to Yasa et al. [20], the position of the condyle is a predictor factor for temporomandibular disorders’ severity. Posteriorly seated condyles were found in patients with severe TMD [21]. However, another study denied this association, suggesting that the posterior condylar position might not be the cause of TMD [22]. Regarding the CBCT assessment of condylar positioning in TMD patients, Paknahad et al. [23] indicated that there is no apparent association between them.

The aim of this study was to evaluate the changes of intra-articular spaces post occlusal splint therapy used in conjunction with cranio-temporomandibular kinesiotherapy in TMD patients.

## 2. Materials and Methods

This retrospective observational study was conducted according to the guidelines of the Declaration of Helsinki and approved by the Institutional Review Board of Iuliu Hațieganu University of Medicine and Pharmacy (no. 403/02.07.2015). Informed written consent was obtained from all subjects involved in the study.

### 2.1. Participants and Intervention

Twenty-four patients (*N* = 24) diagnosed with TMD, according to the Research Diagnostic Criteria for Temporomandibular Disorders (RDC/TMD) [24], were included in this study. Anamnesis, extraoral and intraoral examinations, impressions of both arches (Cavex Cream Alginate, iDent, Chisinau, Republic of Moldova), bite registration using a leaf gauge (Leaf Gauge, Huffman Dental, Kent, OH, USA) and facebow registration (Artex Facebow, Amann Girrbach AG, Koblach, Austria) were performed by an experienced orthodontist (IB). Examinations consisted of inspection and palpation of temporalis and masseter muscles and temporomandibular joint (lateral and posterior pole) in order to assess muscle and joint pain, articular jumps, clicking or crepitus. CBCT examination of both TMJs were performed in a diagnostic imaging center. Inclusion and exclusion criteria for patients enrolled in this study are presented in Table 1.

All the participants underwent occlusal splint therapy and cranio-temporomandibular kinesiotherapy targeting the TMJ and masticatory muscles. Based on facebow mounting (Artex Facebow, Amann Girrbach AG, Koblach, Austria) and bite registration (Occlufast Rock, Zhermack, Badia Polesine, Italy), hard acrylic full coverage occlusal splints with flat occlusal surfaces were manufactured in semi-adjustable articulator (Artex CR, Amann Girrbach AG, Koblach, Austria) in patient’s arc of closure. Heat cured acrylic (Acrylic Vertex Orthoplast, Vertex Dental, Soesterberg, The Netherlands) maxillary occlusal splints divided into three parts (two posterior parts from premolars to molars, one anterior part from canine to canine) were designed. Equal contacts of all mandibular buccal cusps and incisal edges in centric relation and anterior guidance with immediate posterior disocclusion were obtained. Occlusal splints were delivered to all patients. They were advised to wear occlusal splint 24 h/day except for eating and cleaning procedures. Kinesiotherapy was performed by a physiotherapist who specialized in muscles and TMJ manual therapy, including joint mobilization and manipulation and manual therapy of the soft tissues. Kinesiotherapy was indicated to reduce local ischemia, to stimulate proprioception and synovial fluid production and to reduce pain. Additionally, all the patients were trained in and advised to perform Rocabado’s 6 × 6 exercises at home daily. The occlusal splint check-ups and kinesiotherapy took place on a weekly basis until the disappearance of the chief complaint.

All patients underwent a CBCT examination of TMJs before and after the treatment. The primary acquisition was performed using a CBCT device (CRANEX 3D, SOREDEX, Tuusula, Finland) which was set at 6.0–8.0 milliamperage (mA) and 80 kilovoltage (kV); the exposition time was 15 s (s), with a field of view (FOV) of 8 × 5 cm and a slice thickness of 0.3 mm. The scans were performed by the same operator, with patients in sitting positions, holding their heads straight, with Frankfort plane parallel to the ground and the teeth closed in maximum intercuspation. All the examinations respected the ALARA principle (As Low As Reasonably Achievable).

### 2.2. Outcome Measured

The primary outcome was the assessment of the antero-posterior position of mandibular condyles (MC) within the GF post therapy. The patients with TMD were treated with occlusal splints in conjunction with cranio-temporomandibular kinesiotherapy. The secondary outcomes were to evaluate the changes within the distance between the MC and the medial wall of the GF, the glenoid fossa thickness (GFT), the condylar mediolateral width (MLW), and the condylar height (HCo).

Sagittal and coronal slices were used on reconstructed CBCT to measure the intra-articular spaces before and after therapy.

The images were analyzed with OnDemand3DViewer version 1.0 (Cybermed Inc., Seoul, Republic of Korea), by two experienced examiners (MT, AB) trained to perform TMJ linear measurements on the oblique sagittal and coronal reformatted images. All measurements were performed twice with a two-week washout interval, with images being randomly analyzed without access to previous measurements.

**Figure 1:**
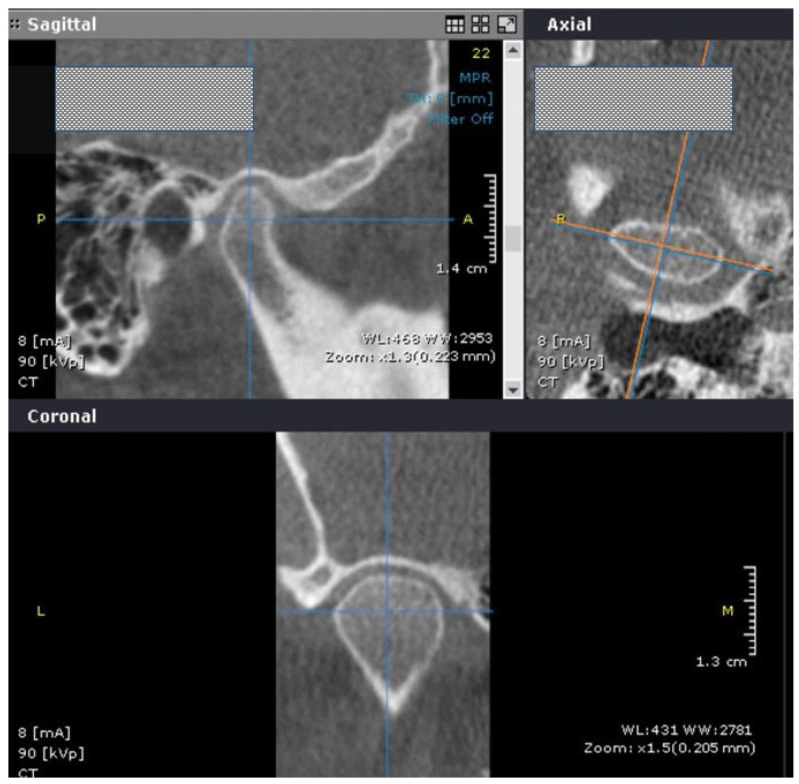
Cone beam computer tomography (CBCT) image in the sagittal, axial, and coronal views showing the slices that were selected for the measurements.

The examiners chose the axial view with the largest mediolateral dimension of the condylar head. Next, a sagittal slice crossing in the middle of the distance between the most prominent points on medial and lateral mandibular condylar poles, and perpendicular to the coronal axis, was selected (Figure 1).

**Figure 2:**
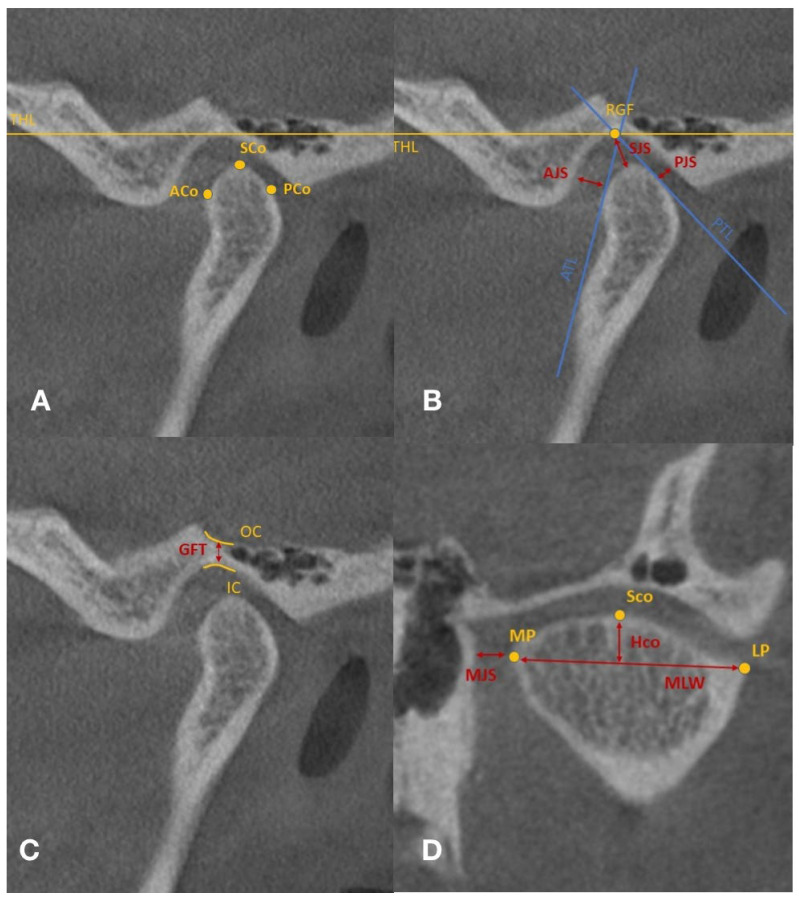
(**A**–**C**) Sagittal slice selected with reference points and lines, measured spaces. (**D**) Coronal slice selected with reference points and lines, measured spaces; ACo—anterior condyle; PCo—posterior condyle; SCo—superior condyle; RGF—roof of glenoid fossa; THL—true horizontal line; ATL—anterior tangent line; PTL—posterior tangent line; AJS—anterior joint space; PJS—posterior joint space; SJS—superior joint space; OC—outer cortical outline; IC—inner cortical outline; GFT—glenoid fossa thickness; MP—medial pole; LP—lateral pole; MJS—medial joint space; MLW—medio-lateral width; HCo—condylar height.

All measurements were performed on the oblique sagittal and coronal reformatted images. The following data were recorded for both TMJs, before and after occlusal splint therapy: anterior joint space (AJS), superior joint space (SJS), posterior joint space (PJS), medial joint space (MJS), condylar medio-lateral width (MLW), glenoid fossae thickness (GFT), and condylar height (HCo) (Table 2 and Figure 2A–D).

The assessment of antero-posterior position of the MC within the GF was performed using Pullinger and Hollender’s formula [25]: Condylar ratio = PJS-AJS/PJS + AJS × 100, where PJS—posterior joint space and AJS—Anterior joint space.

The interpretation of Pullinger and Hollender’s formula according to literature is presented in Table 3.

Based on the condylar ratio changes, the treatment efficacy was assessed. Five percent or more condylar ratio changes were arbitrarily accepted as successful results. A decrease of condylar ratio of 5% or more was considered a negative result. Changes in the condylar ratio of less than 5%, whether they were positive or negative, were considered neutral results according to Derwich et al. [26].

∆ *Condylar ratio (%) = Condylar ratio post treatment (%) − Condylar ratio pre-treatment (%)*

### 2.3. Statistical Analysis

The software used for statistical analysis was R environment for statistical computing and graphics (R Foundation for Statistical Computing, Vienna, Austria), version 4.1.2 [27]. The following parameters were calculated: mean differences, standard deviations (for normally distributed data), medians, upper and lower quartiles (for non-normally distributed data), and ranges. Nevertheless, to help other studies, both types of statistics were used. The assessment of statistical differences before and after the end of treatment was performed using paired *t*-test (for normally distributed data), Wilcoxon’s signed rank test (for non-normally distributed data), and Stuart-Maxwell (for categorical nominal data). To assess intra-observer reliability, the interclass correlation coefficient was computed, along with a 95% confidence interval. The statistical significance level was set at *p* ≤ 0.05. For all the statistical tests we used two-tailed *p* values.

## 3. Results

There were 24 patients diagnosed with TMD included in this study. All the participants (22 women and two men) completed the protocol of treatment. The average age of the participants was 23.88 ± 4.66 years old, with a range between 18 and 33 years old. The main sign was pain in palpation (21/24, 87.5%): muscle pain, joint pain or both. Out of the above mentioned 21 patients, 14 patients (14/21, 66.6%) had associated arthralgia and myalgia pain in palpation. From those 14 patients, eight patients presented disk displacement with reduction (8/14, 57.1%) and one subject presented disk displacement without reduction and crepitus (1/14, 7.1%). Additionally, four subjects (4/21, 19.05%) had arthralgia in palpation alone, and it was associated with disk displacement with reduction in all subjects (4/4,100%). Three subjects (3/21, 14.3%) had myalgia in palpation alone, and only one subject presented disk displacement with reduction (1/3, 33.3%). Additionally, three patients showed no signs of muscle or joint discomfort, although disk displacement with reduction was found (3/24, 12.5%). The main signs and symptoms for the study group are presented in Table 4.

The average number for occlusal splint adjustments was 8.83 times +/− 2.51 with a range between 4 and 14 times. The median time of wearing the occlusal splint was 7 months with an interquartile range between 4.75–10 months. The median kinesiotherapy appointments were 8.5 times with an interquartile range between 6–12.25 times.

Using intraclass-interclass correlation coefficients (ICC) with 95% confidence intervals, Intra-observer reliability for each individual measurement had a range between 0.924 (95% CI: 0.821–0.969) and 0.974 (95% CI: 0.936–0.99). Inter-observer reliability for each individual measurement had a range between 0.826 (95% CI: 0.747–0.881) and 0.981 (95% CI: 0.966–0.989).

Pre- and post-therapy, there were no statistically significant changes in the dimensions of anterior, posterior, superior and medial joint spaces.

Post-therapy, the condylar ratio’s average value increased in a statistically significant manner (*p =* 0.049).

There were no statistically significant changes before and after treatment regarding glenoid fossa thickness (GFT), mediolateral width (MLW) and condylar height (HCo).

Table 5, section A presents the values of measured joint spaces and condylar ratio pre- and post-therapy.

Table 5, section B presents the values of measured osseous structures as thickness of glenoid fossa (GFT), condylar height (HCo) and medio-lateral width (MLW) pre- and post-therapy.

**Figure 3:**
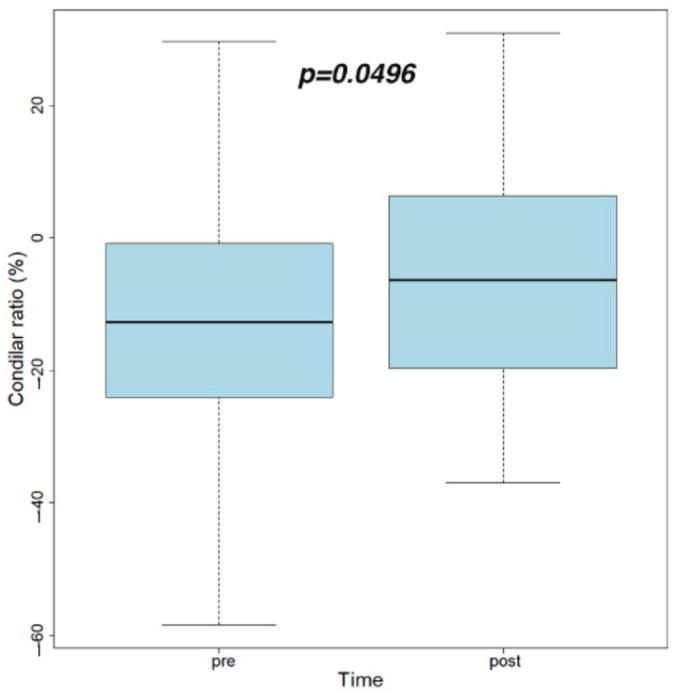
Condylar ratio values pre- and post-treatment and paired *t*-test results.

Figure 3 presents the values of condylar ratio pre- and post-treatment with respectively paired *t*-test results.

Prior to treatment, 24 TMJs were in a posterior position, 18 TMJs were in a central position, and 6 TMJs were in an anterior position within the GF. Post-treatment, 21 TMJs remained posteriorly positioned, 19 TMJs were in a central position and 8 TMJs were in an anterior position. The number of condyles anteriorly positioned increased from 6 (12.5%) to 8 (16.6%). However, the condylar sagittal position pre- and post-treatment had not changed in a statistically significant manner (*p* = 0.649).

Table 6 presents the distribution of condylar sagittal positions within GF pre- and post-treatment.

There were no statistical differences between the right and left TMJs based on condylar ratio changes post-treatment. In 46% of TMJs an increase in the condylar ratio of 5% or more was found. In 23% of TMJs, the condylar ratio was unchanged (Table 7). Asymmetrical TMJ changes were observed in 9 patients (37.5%): 5 patients had an increase in the condylar ratio on the right side and a decrease in the condylar ratio on the left side; 4 subjects had an increase in the condylar ratio on the left side and a decrease in the condylar ratio on the right side.

## 4. Discussions

In this study, the CBCT method was used for assessing the changes in intra-articular spaces and in osseous structures of TMJ. Other radiographic methods for evaluating TMJ include computed tomography (CT) and magnetic resonance imaging (MRI) [28]. However, 3D data acquisition, higher precision and resolution without magnification or distortion, a smaller slice thickness, effective dose, and lower cost compared to CT are some of the reasons why the CBCT is preferred when compared with other imaging techniques for TMJ [29,30]. TMJ soft tissue pathology can still be diagnosed most accurately using MRI; whereas, intra-articular spaces and osseous structures are better evaluated using CBCT [31]. In order to reduce the potential discrepancies in patient positioning during CBCT examination, we used the method recommended by Ikeda et al. [32] in which the image volume was reconstructed in planes parallel and perpendicular to the long axis of the condyle.

Occlusal splint therapy combined with cranio-temporomandibular kinesiotherapy appeared to be effective in pain reduction for TMD patients. Similar results were found by Derwich et al. [26] in a prospective study including 44 TMD patients treated with occlusal splint therapy in conjunction with physiotherapy. This method of therapy, which combines manual therapy and occlusal splints, was more effective in decreasing pain and improving the pressure threshold and dysfunction than the occlusal splint therapy performed alone, according to a study conducted on 16 patients with TMD [33]. Thus, a blind randomized clinical trial on 28 patients with TMD showed that both occlusal splint and manual therapy were effective in pain reduction and in improving mandibular range of motion, especially TMD-related pain [34].

In order to evaluate the antero-posterior position of MC within the GF post-treatment, the anterior, posterior and superior joint spaces were measured on oblique sagittal reformatted images. After treatment, we did not find significant changes in the anterior, posterior, and superior joint spaces in patients with TMD. Furthermore, no significant changes were found in the dimension of the medial joint spaces on the coronal CBCT images. Similar results were found by Derwich et al. [26], even if the linear measurements protocol was different for the medial joint space because it was assessed on an axial CBCT image in the above-mentioned study. Another CBCT study was conducted on 12 TMD patients who underwent occlusal splint therapy followed by occlusal equilibration. Assessment of condylar position of TMD patients by measuring the radiographic joint spaces showed minor changes in condylar position by means of a significant decrease in the anterior space on the left side. The superior and posterior spaces on both sides and the anterior joint spaces on the right side were not statistically changed after treatment [19]. Different results were obtained in a CBCT study on 22 TMD patients using occlusal splints worn 24 h per day for 90 days (except for eating and cleaning procedures): the mean values of the anterior and superior joint spaces significantly increased, respectively; the mean value of the posterior joint space remained nearly unchanged, suggesting a more concentric position of the condyle [35].

The efficacy of occlusal splint therapy combined with kinesiotherapy was assessed based on ∆condylar ratio. According to Derwich et al. [26], an arbitral condylar ratio increase of 5% or more was considered a successful result, while a condylar ratio decrease of 5% or more was considered a negative result. We monitored the efficacy of treatment based on the same formula. In 46% (22/48) of TMJs (11 on right TMJs, respectively, and 11 on left TMJs) an increase of ∆condylar ratio of 5% or more was observed. A percentage of 54% (26/48) of TMJs did not achieve improvement of the value of ∆condylar ratio of at least 5%. Similar results were found by the author mentioned above, respectively; in 53.75% of examined TMJs a ∆condylar ratio of 5% or more was not obtained.

Moreover, asymmetrical TMJ changes were observed in nine patients (37.5%): five patients had an increase in the condylar ratio on the right side and a decrease in the condylar ratio on the left side, respectively, and four subjects had an increase in the condylar ratio on the left side and a decrease in the condylar ratio on the right side. These asymmetrical changes might be correlated with the heterogeneous group of pathologies affecting TMJ, the masticator muscles, or both in TMD patients [23]. However, asymmetrical TMJ changes were not statistically significant between right and left TMJs. Similar asymmetrical TMJ changes between right and left TMJs were found by Derwich et al. [26], but with no statistically significant differences.

Based on Pullinger and Hollender’s formula [25], the condylar ratio was significantly increased after treatment (*p* = 0.049). The condylar ratio increased from an average value of −11.38 to −5.72. Even the difference was statistically significant; both values corresponded to a concentric condylar position, with a tendency, however, to slide anteriorly. The same results were observed by Derwich et al. [26]. This forward movement of MC within the GF to a more centric position could have been due to muscle deprogramming. Assessing qualitative data based on condylar ratio interpretation [25], before the treatment onset, there were 24 (50%) TMJs in a posterior position, 18 (37.5%) TMJs in a central position and six (12.5%) TMJs in an anterior position within the GF. Post-treatment, 21 (43.75%) TMJs remained in a posterior position, 19 (39.5%) TMJs in a central position and eight (16.75%) TMJs in an anterior position. The number of condyles anteriorly positioned increased from six (12.5%) to eight (16.6%) with no statistically significant changes in the condylar sagittal position before and after treatment (*p* = 0.649).

In order to assess osseous structural changes (condylar bone and GF), the thickness of glenoid fossa, the condylar height, and the condyle medio-lateral width all were measured before and after treatment. We did not find any significant changes in the above-mentioned structures. It can be hypothesized that the occlusal splint therapy produced neither condylar remodeling, nor additional degenerative bone changes. A CBCT study in a case–control design found that degenerative bone changes occurred in the subarticular surfaces of the condyle and fossa during TMJ disorders [36]. In our study, osseous changes in joint tissues did not occur during a mean period of seven months of occlusal splint therapy, which may be an indicator for minimally invasive and conservative approach in patients diagnosed with TMD.

A few limitations of this study can be listed. Firstly, the number of participants included in this study was limited. Multicenter studies based on larger groups of patients would be valuable. Moreover, there was no a priori sample size computation for this study. The study group gender ratio (F:M, 22:2) represented another limitation. According to a meta-analysis conducted by Bueno et al. [37], the prevalence of TMD is more than twice as high for females. However, additional equally important factors should also be taken into consideration, such as: self-reported general health conditions, pain disorders, and psycho-emotional factors [38,39]. Moreover, being a retrospective study, the daily wear time of occlusal splint and home exercises were based on patient report. Signs and symptoms, occlusal splint appointments for adjustments, the period of treatment and kinesiotherapy appointments were all accurately reported and registered in the patients’ files. Being a before-after design, any changes were not guaranteed that they were due to the interventions. Additionally, it was difficult to determine which therapy, a combined splint and kinesiotherapy therapy or each of them utilized independently, performed better in relieving pain, especially if used for an extended length of time. A meta-analysis of 52 randomized controlled trials (RCTs) [9] stated that manual therapy (low quality evidence, 83.5%) was the highest-ranked short term (≤5 months) therapy method for muscle pain reduction followed by occlusal splint therapy (moderate-quality evidence, 71.7%). When intermediate term (≥6 months) was considered, the occlusal appliance therapy was the third highest-ranked treatment option for muscle pain reduction (low-quality evidence, 62.8%) after botulinum toxin-A intramuscular injection (very low-quality evidence, 85.8%) and counseling therapy (low-quality evidence, 80%). The age range was limited, with a mean of 23.88 ± 4.66 years old, although only adult patients were included in this study. Manfredini et al. [40] identified in a study conducted on 243 patients seeking TMD treatment that there were at least two distinct age peaks for TMD signs and symptoms: 30–35 years and 50–55 years. Thirdly, all the measurements were performed on CBCT images with a moderate FOV of 8 × 5 cm. A larger FOV would enable the evaluation of additional parameters such as horizontal condylar angle (HCA), which appeared to be increased in a symptomatic TMD group on CBCT scans [41].

The protocol of therapy involved the usage of the occlusal splint continuously, as we had observed that wearing time was correlated with effectiveness of the occlusal splint [8,10].

Even though the currently accepted definition of centric relation in which “condyles articulate in the anterior-superior position against the posterior slopes of the articular eminences” [15], the findings of our study showed that occlusal splint therapy combined with kinesiotherapy decreased the TMD symptoms even if the sagittal condylar position had not changed into the most anterior superior part of the GF.

Further studies should focus on assessing other potential factors in the etiology and treatment of TMD, such as disk positioning, retro discal tissue and cervical spine pathology.

## 5. Conclusions

Occlusal splints therapy combined with cranio-temporomandibular kinesiotherapy in patients with TMD do not change the dimensions of anterior, superior, posterior, and medial TMJ spaces. The sagittal position of the condyle does not change into an anterior-superior position against the posterior slope of articular eminence after treatment. Successful results in terms of pain resolution in TMD patients do not appear to require central condylar positioning.

## Figures and Tables

**Table 1:** Inclusion and exclusion criteria for 24 subjects.

Inclusion Criteria	Exclusion Criteria
Diagnosis of TMD based on RDC/TMD	Age below 18 years old
	History of trauma in head and neck regions
Age above 18 years old	History of previous orthodontic and orthognathic treatment
	Systemic diseases (autoimmune diseases, rheumatoid arthritis)
Willingness to participate in the study	Unwillingness to participate in the study

**Table 2:** List of points and lines used to perform all measurements on sagittal and coronal slices.

Points, Lines, Spaces	Description
Sagital	
ACo (anterior condyle)	Most anterior point of MC
PCo (posterior condyle)	Most posterior point of MC
SCo (superior condyle)	Most superior point of MC
RGF (roof of GF)	Most superior point of GF
THL (true horizontal line)	Horizontal line passing through RGF
ATL (anterior tangent line)	Tangent line from RGF to ACo
PTL (posterior tangent line)	Tangent line from RGF to PCo
AJS (anterior joint space)	Distance between ACo and the posterior slope of articular eminence, measured across the line, which is perpendicular to ATL
PJS (posterior joint space)	Distance between PCo and the posterior wall of GF, measured across the line, Pwhich is perpendicular to PTL
SJS (superior joint space)	Distance between RGF and SCo
OC (outer cortical outline)	Outer cortical of GF
IC (inner cortical outline)	Inner cortical of GF
GFT (glenoid fossa thickness)	Distance between OC and IC
Coronal	
MP (medial pole)	Most prominent point of MP of MC
LP (lateral pole)	Most prominent point of LP of MC
MJS (medial joint space)	Distance between MP and medial wall of GF
MLW (medio-lateral width)	Distance between MP and LP
HCo (condylar height)	Distance between SCo and MLW, measured across a line which is perpendicular to a line connecting MP and LP

**Table 3:** Interpretation of formula presented by Pullinger and Hollender.

Condylar Ratio	Interpretation
0 ± 12%	Central position of MC within GF
<−12%	Posterior position of MC within GF
>+12%	Anterior position of MC within GF

MC—mandibular condyle.

**Table 4:** Signs and symptoms for the study group.

	Main Symptoms	Associated Signs
Pain in palpation (21/24, 87.5%)	arthalgia and myalgia (14/21, 66.6%)	DDWR (8/14, 57.1%)
		DDwoR and crepitus (1/14, 7.1%)
	arthalgia (4/21, 19.05%)	DDWR (4/4, 100%)
	myalgia (3/21, 14.3%)	DDWR (1/3, 33.3%)
	Associated signs	
No pain in palpation (3/24, 12.5%).	DDWR (3/3, 100%)	

DDWR—disk displacement with reduction; DDwoR—disk displacement without reduction.

**Table 5:** The average values for measured joint spaces and osseous structures, respectively for condylar ratio pre- and post-treatment.

Time:	Pre	Post	*p*-Value
	(*n* = 48)	(*n* = 48)	
Section A			
AJS (mm)			
median (Q1, Q3)	2.25 (1.88–2.58)	2.2 (1.9–2.6)	
Av. (SD)	2.38 (0.71)	2.25 (0.61)	0.121 ^a^
Range	1.35–4.88	1.20–3.70	
PJS (mm)			
median (Q1, Q3)	1.81 (1.48–2.27)	1.95 (1.55–2.4)	
Av. (SD)	1.90 (0.63)	2.02 (0.61)	0.277 ^a^
Range	0.80–4.16	1.14–3.51	
SJS (mm)			
median (Q1, Q3)	2.67 (2.02–3.29)	2.62 (1.9–3.26)	
Av. (SD)	2.71 (0.83)	2.63 (0.88)	0.774 ^a^
Range	1.35–4.47	1.25–4.54	
MJS (mm)			
median (Q1, Q3)	3.29 (2.81–3.75)	2.95 (2.38–4.01)	
Av. (SD)	3.45 (1.13)	3.26 (1.16)	0.176 ^a^
Range	1.32–7.21	1.05–6.47	
Condylar ratio (%)			
HCo	−12.68 (−23.62–−0.95)	−6.31(−19.57–5.97)	
Av. (SD)	−11.38 (18.80)	−5.72 (19.13)	0.0496 ^b^
Range	–58.36–29.70	−36.96–30.86	
Condylar position			
Posterior (condylar ratio < −12%)	24 (50)	21 (43.75)	0.649 ^c^
Central (−12% < condylar ratio ≤ 12%)	18 (37.5)	19 (39.58)	
Anterior (condylar ratio > 12%)	6 (12.5)	8 (16.67)	
Section B			
GFT (mm)			
median (Q1, Q3)	1.16 (0.79–1.89)	1.07 (0.78–1.59)	0.473 ^a^
Av. (SD)	1.46 (1.15)	1.39 (1.10)	
Range	0.25–7.67	0.1–5.96	
HCo (mm)			
median (Q1, Q3)	4.97 (4.29–5.86)	5.17 (4.14–5.79)	0.204 ^b^
Av. (SD)	5.19 (1.26)	5.03 (1.16)	
Range	2.88–8.37	2.49–6.95	
MLW (mm)			
median (Q1, Q3)	17.65 (15.87–19.23)	17.59 (16.14–19.08)	
Av. (SD)	17.45 (2.44)	17.54 (2.23)	0.661 ^b^
Range	11.21–21.42	11.95–21. 97	

^a^ Wilcoxon’s signed rank test; ^b^ paired *t*-test, ^c^ Stuart-Maxwell test; Av.—average; SD—standard deviation; Q1—lower quartile; Q3—upper quartile; AJS—anterior joint space; PJS—posterior joint space; SJS—superior joint space; MJS—medial joint space; GFT—glenoid fossa thickness; HCo—condylar height; MLW—mediolateral width.

**Table 6:** The distribution of condylar sagittal position within the GF pre- and post-treatment.

Condylar Ratio Interpretation Pre- Treatment	Condylar Ratio Interpretation Post- Treatment (%)
	Anterior: 2 (33.33%)
Anterior (*n* = 6) = >	Central: 4 (66.67%)
	Posterior: 0 (0%)
	Anterior: 4 (22.22%)
Central (*n* = 18) = >	Central: 9 (50%)
	Posterior: 5 (27.78%)
	Anterior: 2 (8.33%)
Posterior (*n* = 24) = >	Central: 6 (25%)
	Posterior: 16 (66.67%)
*p* = 0.649 ^a^	

^a^ Stuart-Maxwell test.

**Table 7:** The treatment outcome assessment based on condylar ratio changes pre- and post-treatment (∆Condylar ratio = Condylar ratio post-treatment − Condylar ratio pre-treatment).

	Left TMJ	Right TMJ	*p*-Value
	(*n* = 24)	(*n* = 24)	
∆ Condylar ratio (%)	negative: 8 (33.33)	negative: 7 (29.17)	0.924 ^a^
	neutral: 5 (20.83)	neutral: 6 (25)	
	positive: 11 (45.83)	positive: 11 (45.83)	

^a^ chi square test.
